# Supplementary material for: Enhanced inter-regional coupling of neural responses and repetition suppression provide separate contributions to long-term behavioral priming
Source: Commun Biol. 2021 Apr 20;4:487. doi: 10.1038/s42003-021-02002-7 (PMC8058068; doi:10.1038/s42003-021-02002-7)
Supplement: Supplementary file 2 — Supplementary information [file 42003_2021_2002_MOESM2_ESM.pdf]

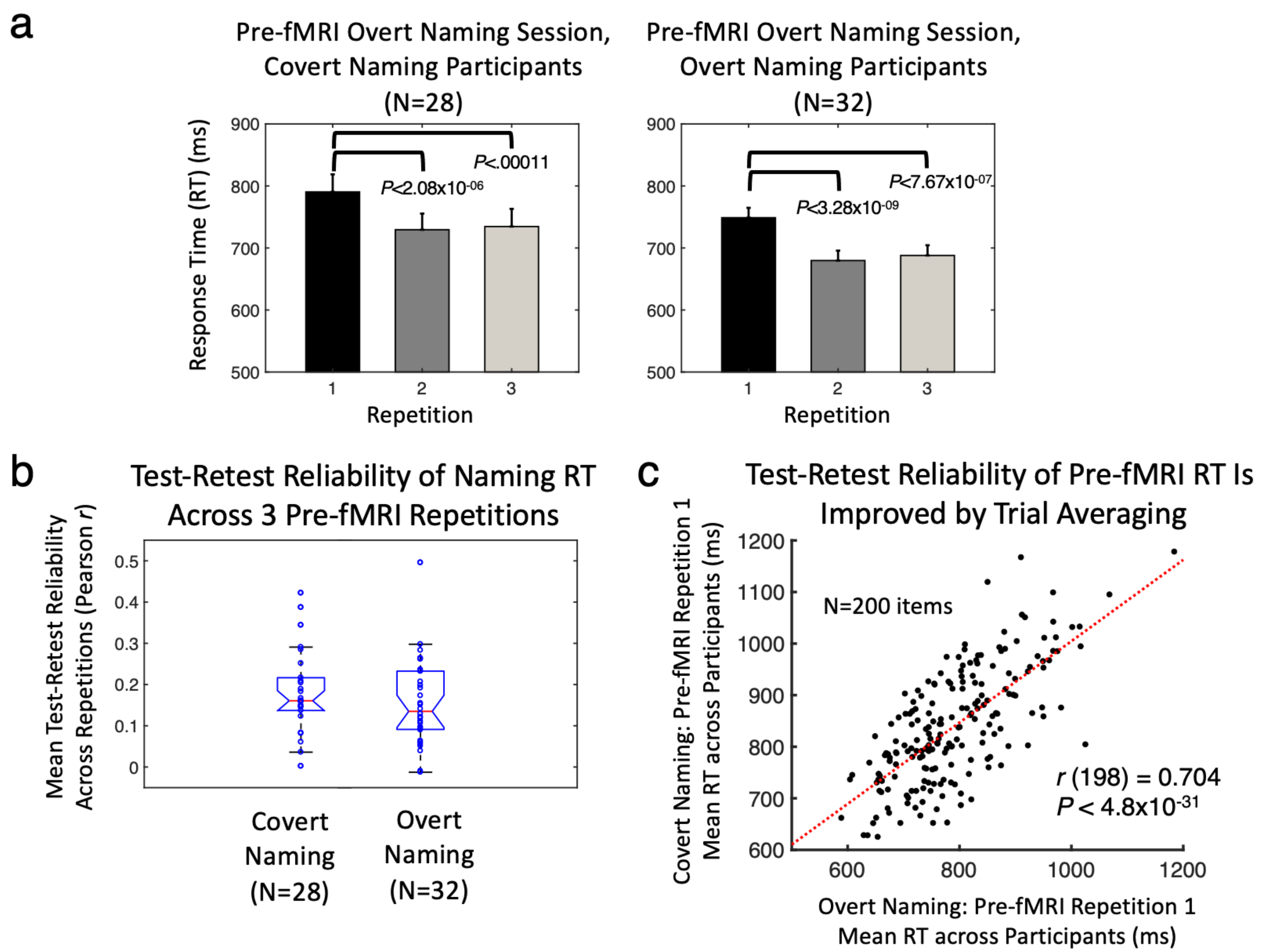

Supplementary Figure 1. Individual Picture Naming Response Times are Highly Variable but Become Reliable when Averaged. Test-retest reliability of individual response times (RT) was evaluated using the pre-fMRI Overt Naming sessions for participants performing both Overt (N=32) and Covert Naming (N=28) conditions during fMRI. (a) Each participant named a set of 100 pictures presented 3 times in a pseudorandom order in a quiet testing room prior to fMRI (Repetitions 1-3). As expected, decreased RTs were observed on the 2nd and 3rd repetitions relative to the 1st presentation in both sets of participants (calculated on correct naming trials). Error bars indicate standard error of the mean (SE). (b) Test-retest reliability (Pearson *r*) was calculated for each participant using the 3 pre-fMRI repetitions (1st vs 2nd, 2nd vs 3rd, 1st vs 3rd), including only items named correctly for all 3 repetitions (a mean of 80.48 items per participant out of a possible 100). The distributions of mean reliability (over all combinations of repetition pairs) across all participants is shown for Overt and Covert Naming participants separately using boxplots, along with all individual participant datapoints as open circles. The red horizontal line in each box plot represents the median (50th %-ile), the blue horizontal lines just above and below the median represent the 25th and 75th %-iles, the black horizontal lines enclose +/- 2.7 standard deviations of the mean (99.3% coverage of a normal curve), and the boundaries of the horizontal notches inside the 25th and 75th %-iles depict the 95% confidence limits of the median. The distributions do not differ significantly between the Overt and Covert Naming participants (median Covert reliability = 0.1607; median Overt reliability = 0.1350; overall median = 0.1567). (c) Despite the poor test-retest reliability of the individual RTs, averaging RTs across participants by item (or across items within-participant; see Figure 4C) leads to large improvements in reliability. Shown is the test-retest reliability (Pearson *r*) when averaging across participants in each task condition by item (Overt vs Covert). As pre-exposed sets were counter-balanced across participants, averaging included slightly less than half of the number of participants in each condition (correct responses only; median number of participants averaged per item was 15 for Overt Naming, 11 for Covert Naming). After averaging over participants and only utilizing RTs from Repetition 1 (the first time each participant encountered an item), the test-retest reliability of item RTs increases to 0.704. These results suggest that successfully identifying the neural correlate of repetition priming will require a degree of trial averaging/pooling; i.e. measuring the change in RT on individual trials and relating that to the change in single-trial BOLD responses will not be practical, even if only considering the reliability of behavior. Related to Figure 4.

Agreement of Repetition X Primeability Interaction Across Tasks

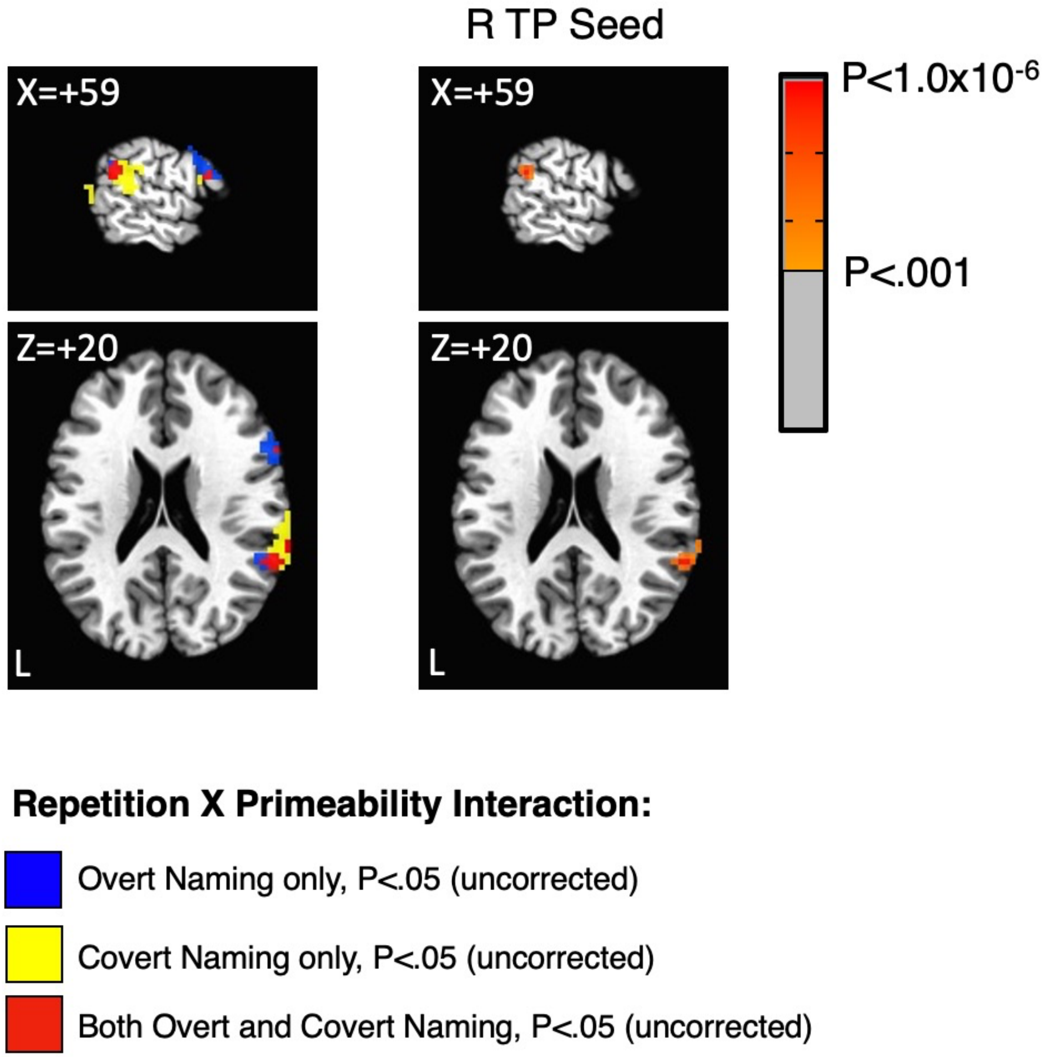

Supplementary Figure 2. Agreement of Repetition X Primeability Interaction in Seed Detection Across Tasks. The Repetition X Primeability interaction in whole-brain connectedness found in the right temporoparietal (R TP) cortex failed to interact significantly with Task (Overt vs Covert Naming). Shown in the left panels are voxels that are significant in each task individually (P<.05, uncorrected) (blue = Overt Naming only; yellow = Covert Naming only; red = significant in both). These results were masked by voxels that showed a consistent interaction pattern across both tasks (P<.05, uncorrected, when including data from both tasks; minimum cluster size of 100 voxels). Virtually all of the voxels in the R TP ROI surviving whole-brain correction in the full data (Figure 5A, shown for reference in right panels) were significant at an uncorrected level in both tasks individually. A cluster in the right frontal cortex corresponded to a cluster in the full analysis that failed to survive whole-brain correction, but showed a similar pattern to that in R TP. The locations of both the R TP and right frontal clusters agree well with the locations of clusters in the Ventral Attention network[92]. Related to Figure 6.

Increased Coupling Occurs with No Increase in Activity Levels

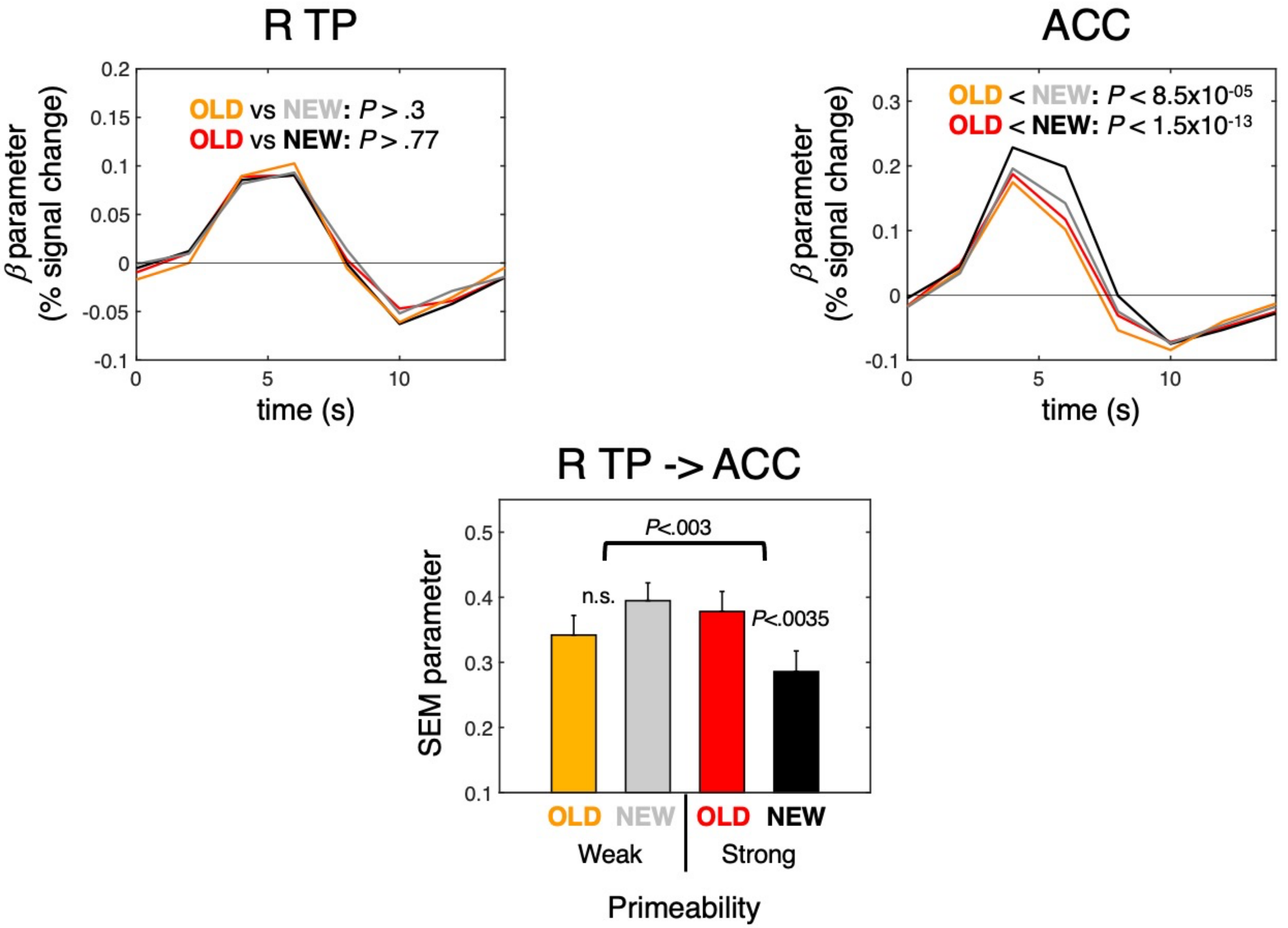

Supplementary Figure 3. Increased coupling occurs with no increase in activity levels. The increased coupling observed from the R TP to ACC ROIs, particularly in the Strong Primeable condition (see bottom panel), happens despite no increase in activity levels in R TP (Strong Primeable, OLD vs NEW: $P>.77$) and a decrease in activity in ACC (Strong Primeable, OLD < NEW: $P<1.5\times10^{-13}$). In the absence of a change in the synchronization of the underlying activity in R TP and ACC, increased synaptic coupling onto ACC would be expected to lead to higher, not lower activity in ACC. The fact that activity is not increased in R TP, coupling from R TP is increased to ACC, and activity is decreased in the ACC is most consistent with increased synchronization of neural activity one or both regions simultaneously with the increased interregional coupling (see [93] for related computational modeling and discussion). The repetition suppression in the ACC is itself largely unrelated to the coupling increases, as detailed in the partial correlation analysis in the main text. Related to Figure 7.

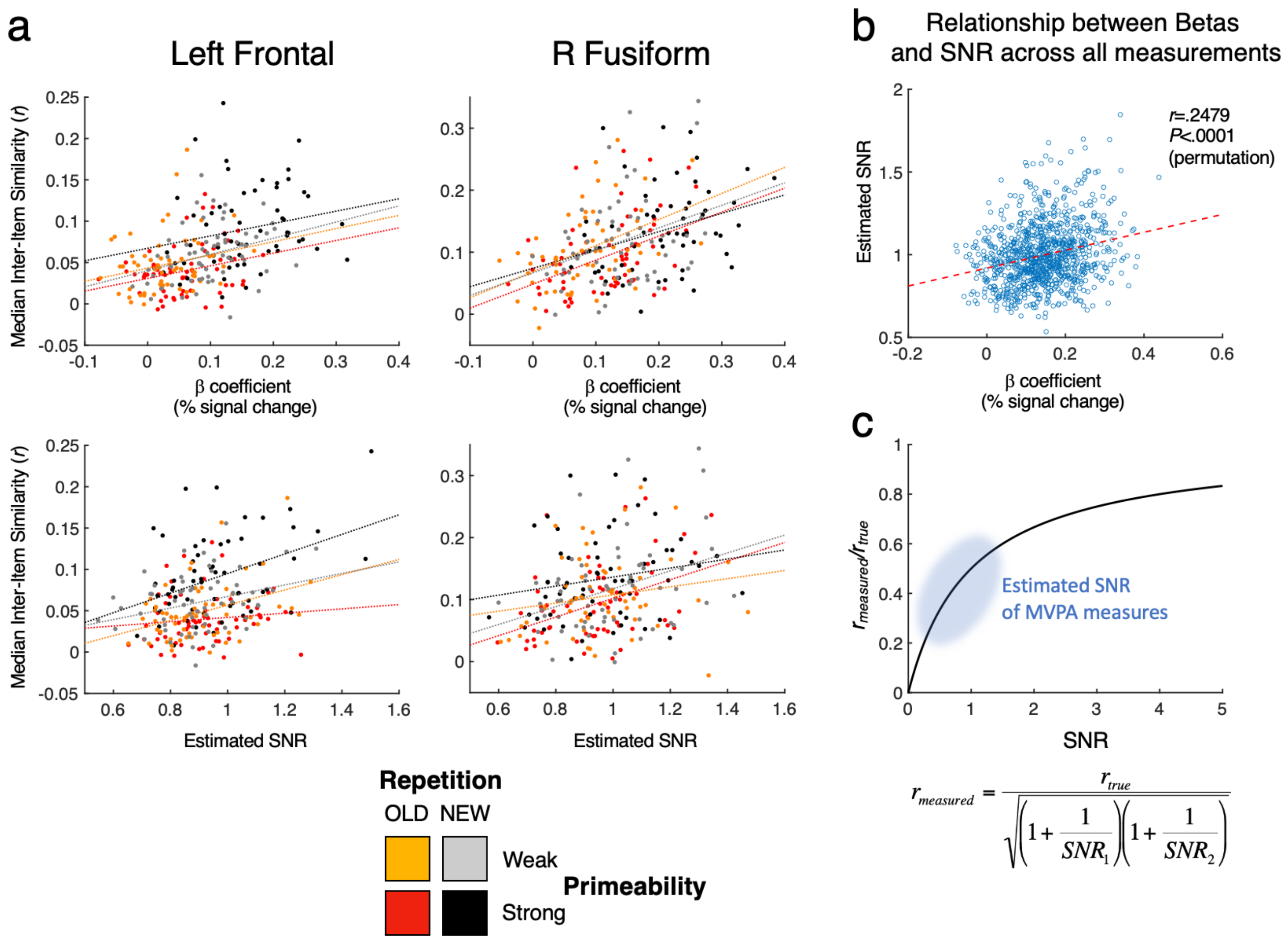

Supplementary Figure 4. Similarity measures calculated on single-trial BOLD responses are strongly related to average activity level and signal-to-noise ratio. (a) The dependence of similarity (Pearson *r*; on y axes) on beta values and estimated SNR (x axes) are shown more directly using scatterplots. In each scatterplot, each participant contributes 4 datapoints, one for each experimental condition (Repetition X Primeability; see color key at bottom). Each scatterplot shows 4 best-fit lines, one for each condition's points separately. If variability along the y axis is due only to the corresponding nuisance factor, then all points should lie along a single best fit line with increasing slope. However, even if there is a strong effect of the nuisance factor, significant shifts along the y axis can indicate a real, residual effect of condition. The results for the R Fusiform ROI in the right panels are consistent with all differences in similarity being due to the nuisance factors (note the highly similar best-fit lines, all with positive slope). Results for the Left Frontal ROI are less definitive, with a possible vertical shift in the position of the Strong Primeable, NEW fit line. However, it is also possible that the best-fit lines are simply noisy. The loss of findings for all other conditions in other ROIs suggests that SNR-level is poor enough that these results are, at best, indeterminate. (b) As anticipated, the average beta coefficient is related to estimated SNR, with beta values closer to zero associated with the lowest SNRs. The scatterplot shows all beta/SNR datapoints across all participants and ROIs, with significance testing by permutation (shuffling which beta goes with which SNR measure; 20,000 iterations). (c) The estimated SNRs from the current experiment lie in the range of 0.5 to 1.5, with a mean of approximately 1 (indicating equal levels of signal and noise). Given the likely presence of noise in the numerator of the calculations (see Online Methods), it is important to emphasize that these estimates are upper-bound estimates, which could mean the SNRs are actually poorer. In the plot, the estimated SNRs for the current MVPA data are shown relative to the theoretical curve of SNR-related attenuation of Pearson correlation coefficients (expressed as a fraction of the true correlation, e.g. 0.5; SNR on the x-axis actually depends on the individual SNRs of both patterns being correlated – see equation -- but is assumed to be the same for the two patterns here for simplicity). At SNRs of 1.0, small changes in SNR yield large differences in correlation (ranging from 0.2 to 0.6 of the true value when changing from SNRs of .5 to 1.5). At weaker values of SNR, this slope is even steeper. Taken together, it is possible that low beta weights lead to low SNR, which leads to low pattern similarity measures, potentially explaining the full pattern of raw similarity measures in Figure 8A. The level of SNR in single-trial fMRI responses may simply be too low to evaluate the Sharpening model in fMRI. Averaging responses across several repetitions, as is commonly done, is also inappropriate in this case, as testing the Sharpening model requires an estimation prior to any repetition. Related to Figure 8.

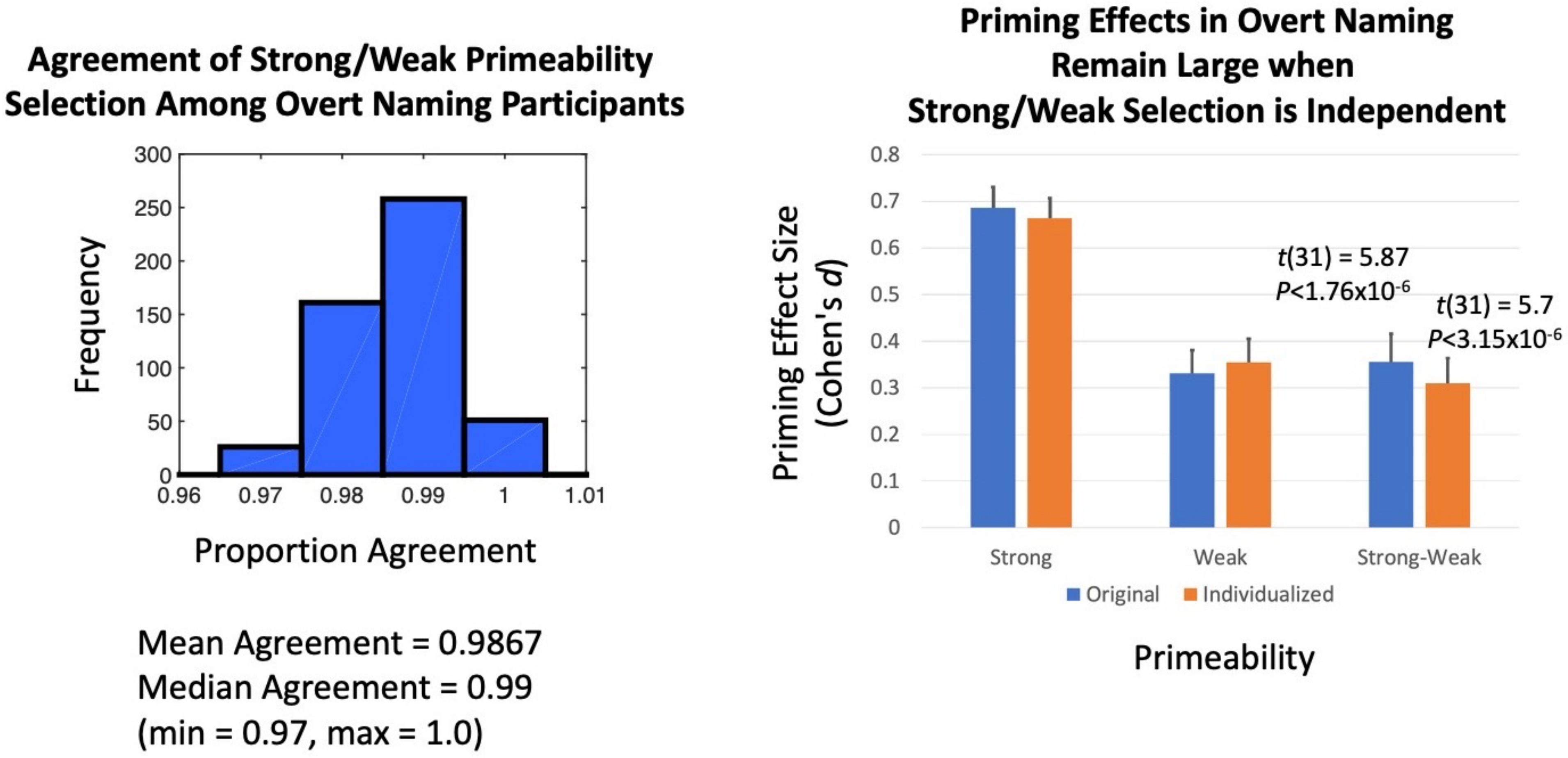

Supplementary Figure 5. Effect of leaving out each Overt Naming participant's fMRI behavioral data on Strong/Weak Primeability selection. Left panel shows the proportion agreement among Overt Naming participants when the selection of Strong versus Weak Primeability is individualized by excluding each participant's data for their own Strong/Weak selection. Frequency histogram is tabulated across all possible pairings of participants. Right panel shows that the priming magnitudes in Strong versus Weak conditions continue to be quite large when these individualized item selections are used (orange compared to blue bars).

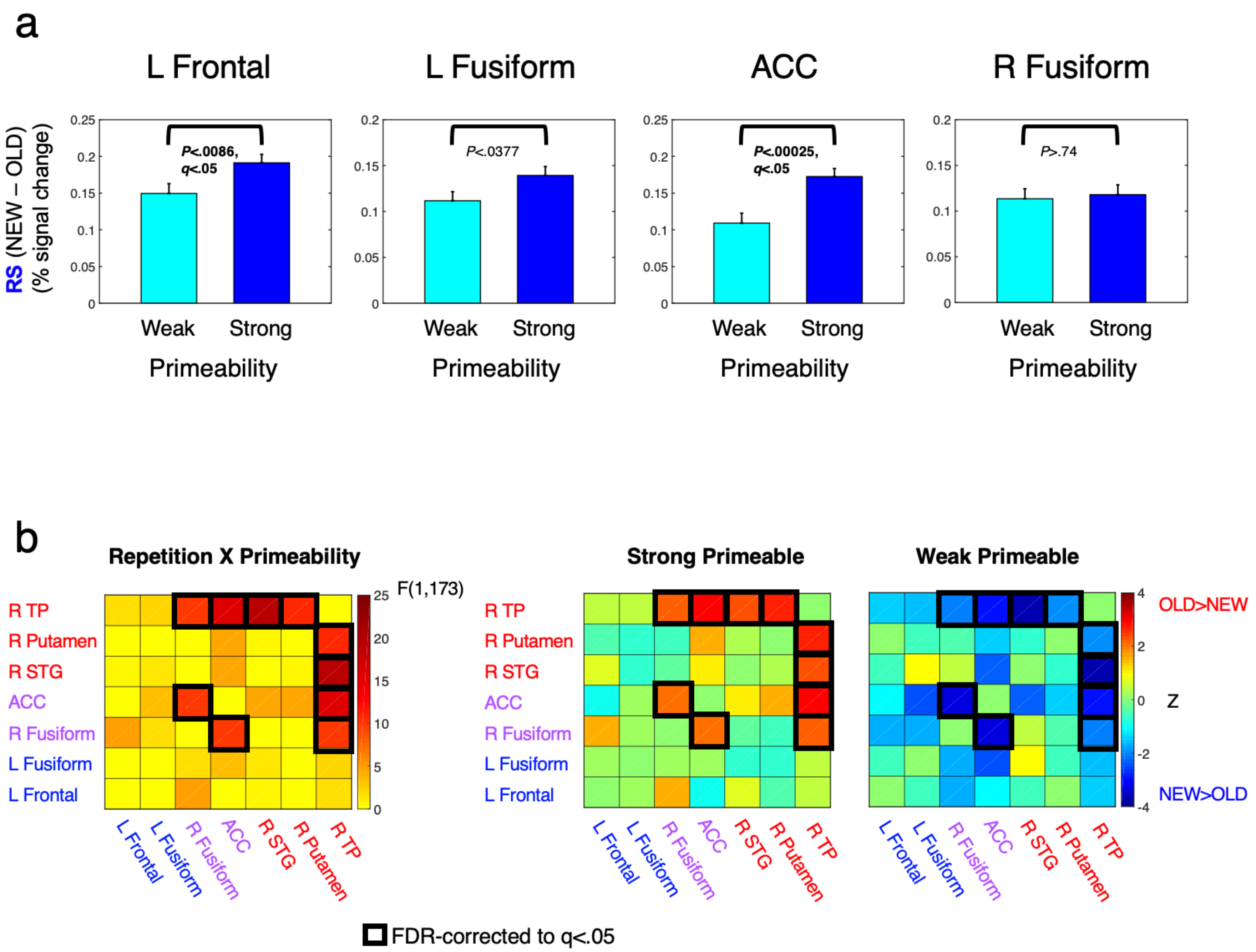

Supplementary Figure 6. Effect of independent Strong/Weak Primeability selection on RS and Functional Connectivity effects. (a) Weak versus Strong Primeability effects in RS are qualitatively similar with independent selection of Strong/Weak for all participants. Only the effects in L Fusiform fail to survive correction for multiple comparisons by FDR (results in Figure 5a, which were unaffected by Strong/Weak selection, also included in FDR calculation). (b) Functional connectivity effects are qualitatively unchanged and remain significant and corrected by FDR when Strong/Weak selection is independent for all participants (compare to Figure 5 and Figure 6c).

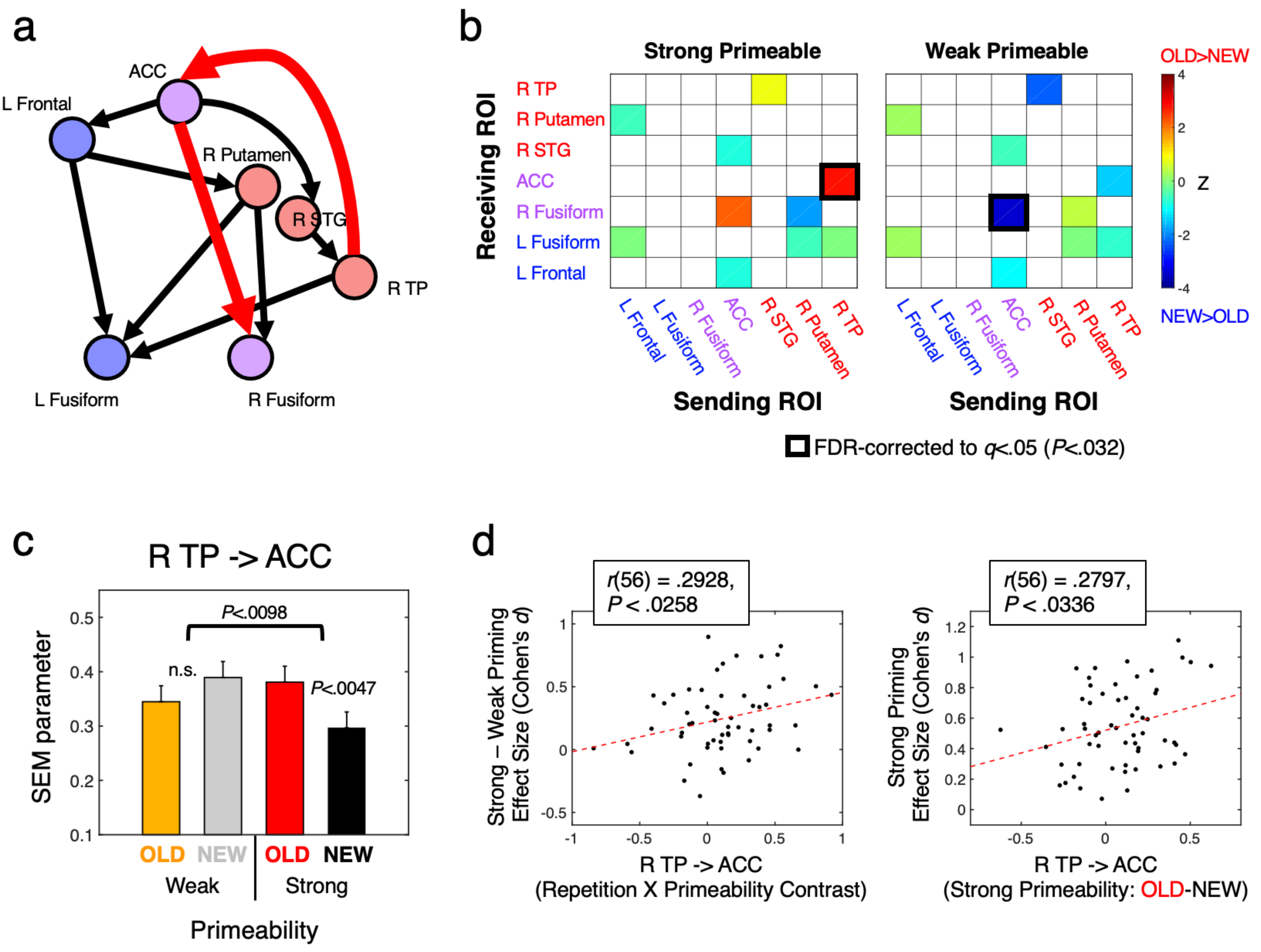

Supplementary Figure 7. Effect of independent Strong/Weak Primeability selection on Effective Connectivity effects. (a-c). Effective connectivity effects are qualitatively similar, with 2 of the 3 original Strong/Weak X OLD/NEW interactions remaining significant and corrected by FDR (q<.05). (d) Brain-behavioral correlations involving the R TP->ACC connection and supporting the predictions of the Synchrony model also remain significant (compare to Figure 7a-d).

	ROI Label	Peak Coordinate (Talairach-Tournoux) X	Y	Z	Spatial Extent RS effect (mm^3)	Spatial Extent FC effect (mm^3)	Overlap (mm^3)
1	Left Frontal	-40	+5	+26	11934		
2	Left Fusiform Gyrus	-40	-49	-10	5940		
3	Right Fusiform Gyrus*	+41	-40	-16	4455	1080	189
4	Anterior Cingulate (ACC)*	+5	+2	+50	4752	891	324
5	Right STG	+44	-37	+11		918	
6	Right Putamen	+29	-16	+8		837	
7	Right Temporoparietal	+59	-43	+20		756	

* - Peak Coordinate used was from FC effect (in overlapping voxels with RS effect)

Supplementary Table 1. Regions of Interest. Regions of interest used in Functional (FC) and Effective Connectivity (EC) Analyses (Repetition Suppression, RS, and FC regions), as well as subsequent tests of Facilitation and Sharpening models (RS regions only). Standard space coordinates (Talairach-Tournoux) of the peak statistic used to identify effects are reported, along with the spatial extent of the associated clusters by effect type. For Right Fusiform and ACC ROIs, detected in both RS and FC analyses, the peak coordinate used is related to the peak of the FC effects within the voxels that overlapped across the two effects (in order to maximize the observation of any FC/EC effects). Full ROI-ROI matrix analyses of FC/EC effects, as well as tests of the Facilitation model, used spherical ROIs centered on the peak coordinates. MVPA tests of the Sharpening model used the full spatial extent of RS clusters. Related to Figure 6.

		Current Study: Per Item Measures	
		Priming Magnitude (NEW-OLD)/NEW RT	Naming RT (NEW)
**Recognition Memory Measures Per Item (Gilmore et al., 2019)**	Corrected Hit Rate (Hits - False Alarms)	0.0819	-0.0927
	(NEW-OLD)/NEW RT	-0.141	0.0087

Supplementary Table 2. Priming Magnitude and Naming Performance Per Item are Not Strongly Related to Recognition Memory Performance on Same Items. Priming magnitudes and Naming response times (RT) per item (N=200 pictures) from the current study were correlated (Pearson) with Recognition Memory Performance per item on the same stimuli in Gilmore et al. (2019) with N=40 different participants. Recognition memory performance measures include both Corrected Hit Rate (Hit Rate - False Alarm Rate) and an RT-based measure of recognition memory strength: [(NEW-OLD)/NEW] RT. The lack of correlation with Naming RT, which was used to split Strong and Weak Primeability, suggests that Primeability is not strongly related to Recognition Memory (sharing < 1% of the total variance in either measure).
