# Supplementary material for: Enhanced inter-regional coupling of neural responses and repetition suppression provide separate contributions to long-term behavioral priming
Source: Commun Biol. 2021 Apr 20;4:487. doi: 10.1038/s42003-021-02002-7 (PMC8058068; doi:10.1038/s42003-021-02002-7)
Supplement: Supplementary file 3 — Reporting summary [file 42003_2021_2002_MOESM3_ESM.pdf]

nature research

Corresponding author(s): Stephen J. Gotts, PhD (gottss@mail.nih.gov)

Last updated by author(s): 3/11/2021

Reporting Summary

Nature Research wishes to improve the reproducibility of the work that we publish. This form provides structure for consistency and transparency in reporting. For further information on Nature Research policies, see our Editorial Policies and the Editorial Policy Checklist.

Statistics

For all statistical analyses, confirm that the following items are present in the figure legend, table legend, main text, or Methods section.

n/a	Confirmed	
☐	☒	The exact sample size (*n*) for each experimental group/condition, given as a discrete number and unit of measurement
☐	☒	A statement on whether measurements were taken from distinct samples or whether the same sample was measured repeatedly
☐	☒	The statistical test(s) used AND whether they are one- or two-sided<br>*Only common tests should be described solely by name; describe more complex techniques in the Methods section.*
☐	☒	A description of all covariates tested
☐	☒	A description of any assumptions or corrections, such as tests of normality and adjustment for multiple comparisons
☐	☒	A full description of the statistical parameters including central tendency (e.g. means) or other basic estimates (e.g. regression coefficient) AND variation (e.g. standard deviation) or associated estimates of uncertainty (e.g. confidence intervals)
☐	☒	For null hypothesis testing, the test statistic (e.g. *F*, *t*, *r*) with confidence intervals, effect sizes, degrees of freedom and *P* value noted<br>*Give P values as exact values whenever suitable.*
☐	☒	For Bayesian analysis, information on the choice of priors and Markov chain Monte Carlo settings
☐	☒	For hierarchical and complex designs, identification of the appropriate level for tests and full reporting of outcomes
☐	☒	Estimates of effect sizes (e.g. Cohen's *d*, Pearson's *r*), indicating how they were calculated

Our web collection on statistics for biologists contains articles on many of the points above.

Software and code

Policy information about availability of computer code

Data collection	MRI data were collected on a GE 3T scanner. Behavioral data were acquired outside the MR scanner with Presentation Ver 11.3. Inside the MRI scanner, spoken responses were captured with an Opto-Acoustics FOMRI-III NC MR-compatible microphone with built-in noise cancellation and routed into an M-Audio FastTrack Ultra 8-R USB audio interface. Responses were recorded with Adobe Audition. To calculate response times, the stimulus presentation computer emitted a square wave pulse at the onset of each trial and a custom Matlab program calculated the time difference between the square pulse onset and voice response onset for each trial.
Data analysis	The AFNI software suite of programs for fMRI analyses was used, with custom csh scripts used to organize program calls. All GLM, functional connectivity and effective connectivity used the respective AFNI programs listed in the methods, with 3dLME (linear mixed effects models) used for most analyses. Cohen's d for behavioral priming effect sizes was calculated for each participant in Excel spreadsheets from the raw RTs. Functional/effectivity connectivity results were prepared for display in Matlab, and correlations and partial correlations calculated in scatterplots were also conducted in Matlab. False Discovery Rate corrections in matrix analyses were calculated in Matlab (using code available from Prof Tom Nichols @ Oxford Univ). Continuous hemodynamic response function best fits to TR-specific beta coefficients were calculated in custom Matlab code. All code is available online at figshare.com.

For manuscripts utilizing custom algorithms or software that are central to the research but not yet described in published literature, software must be made available to editors and reviewers. We strongly encourage code deposition in a community repository (e.g. GitHub). See the Nature Research guidelines for submitting code & software for further information.

Data

Policy information about availability of data

All manuscripts must include a data availability statement. This statement should provide the following information, where applicable:

- Accession codes, unique identifiers, or web links for publicly available datasets
- A list of figures that have associated raw data
- A description of any restrictions on data availability

Our data availability statement is included on p.44 of the manuscript. The protocol used at the time of data collection did not provide for automatic data sharing (all current protocols have this). Our statement: "Raw MRI data are available via the XNAT platform. Users will need to request access through the XNAT system (https://central.xnat.org/app/template/Index.vm). This can be done by creating an XNAT user account and pressing the 'request access' link. Processed, standard-space (Talairach) fMRI data (NIfTI format) and behavioral (response time) data are available from the authors (SJG) on request. All data used to create the figures in the main text is available at Figshare (https://figshare.com/projects/Gotts_Milleville_Martin_2021_Communications_Biology_/99527)."

Field-specific reporting

Please select the one below that is the best fit for your research. If you are not sure, read the appropriate sections before making your selection.

☐ Life sciences ☒ Behavioural & social sciences ☐ Ecological, evolutionary & environmental sciences

For a reference copy of the document with all sections, see nature.com/documents/nr-reporting-summary-flat.pdf

Behavioural & social sciences study design

All studies must disclose on these points even when the disclosure is negative.

Study description	This study is quantitative experimental.
Research sample	The research sample is composed of NIH/NIMH healthy volunteers. Thirty-two participants performed the Overt Naming Task (14 males, 18 females) with a mean (SD) age of 24.03 (3.58) years (range: 19 to 38), and 28 additional participants performed the Covert Naming Task (9 males, 19 females) with a mean (SD) age of 23.43 (1.62) years (range: 21 to 28). Participants were right-handed, neurologically healthy native English speakers with normal or corrected-to-normal vision. The characteristics of this study sample are typical of this study topic (usually neurotypical participants of undergraduate or graduate student age who participate in university subject pools).
Sampling strategy	All participants received all experimental conditions within a given task condition (within-participant) and task was sampled consecutively from the subject pool (first Overt Naming, then Covert Naming). The desired sample size per task was approximately 30, as previous studies attempting to analyze correlations between functional connectivity and behavior using Pearson's r have required in the range of 30-40 participants (for brain behavioral correlations in the range of 0.3 to 0.4, the needed sample size to reach P<.05 ranges from 25-44). For effects that are common across Overt and Covert Naming, it was also thought that around 60 participants would then be available for the examination of behavioral correlations.
Data collection	MRI data were logged automatically on the dedicated image recon server in the NIH MR Center. Verbal responses were recorded as .wav files in a continuous stream throughout each experimental run (with trial onset recorded in parallel as a trigger pulse, for later response time calculation). The experimenter scored picture naming correctness (and transcribed error responses) online during the session. Participant head motion was also monitored online during each run with AFNI RealTime, with feedback provided to the participant.
Timing	Data collection began August 2012 and continued through April 2015.
Data exclusions	4 participants were excluded from the Overt Naming task: 2 had abnormal clinical scans (excluded per protocol), 1 participant for display computer malfunction (data were only available for 2/5 runs), and 1 participant for excessive motion (> 0.3 mm/TR on 2/5 runs). We have used this same motion cutoff in prior studies.<br><br>3 participants were excluded from the Covert Naming task: 1 participant for failing to follow task instructions during fMRI (button press times too fast to be real: mean RT ~ 300 ms), 2 participants for display computer malfunction affecting > 2 runs.<br><br>2 participants were excluded from Effective Connectivity analyses, as SEM failed to find parameters for the optimal model for at least one of their experimental conditions.
Non-participation	No participants dropped out or declined participation.
Randomization	All participants received all experimental conditions within a given task condition (within-participant) and task was sampled consecutively from the subject pool (first Overt Naming was completed, then Covert Naming).

Reporting for specific materials, systems and methods

We require information from authors about some types of materials, experimental systems and methods used in many studies. Here, indicate whether each material, system or method listed is relevant to your study. If you are not sure if a list item applies to your research, read the appropriate section before selecting a response.

Materials & experimental systems

n/a	Involved in the study
☒	☐ Antibodies
☒	☐ Eukaryotic cell lines
☒	☐ Palaeontology and archaeology
☒	☐ Animals and other organisms
☐	☒ Human research participants
☒	☐ Clinical data
☒	☐ Dual use research of concern

Methods

n/a	Involved in the study
☒	☐ ChIP-seq
☒	☐ Flow cytometry
☐	☒ MRI-based neuroimaging

Human research participants

Policy information about studies involving human research participants

Population characteristics	Thirty-two participants performed the Overt Naming Task (18 females) with a mean (SD) age of 24.03 (3.58) years (range: 19 to 38), and 28 additional participants performed the Covert Naming Task (19 females) with a mean (SD) age of 23.43 (1.62) years (range: 21 to 28). Participants were right-handed, neurologically healthy native English speakers with normal or corrected-to-normal vision.
Recruitment	Participants were enrolled for experiment participants as part of our larger laboratory protocol (all of NIMH Laboratory of Brain and Cognition, protocol 93-M-0170, clinical trials number NCT00001360). Participants are paid for participation and publicly recruited from the local community.
Ethics oversight	Ethics approval for this study was granted by the NIH Institutional Review Board (protocol 93-M-0170, clinical trials number NCT00001360).

Note that full information on the approval of the study protocol must also be provided in the manuscript.

Magnetic resonance imaging

Experimental design

Design type	Quasi-slow-event related (not waiting a full 16 seconds between trials for HRF recovery, but long enough to separate the peak BOLD responses; trial durations 8-14 seconds in multiples of the 2-sec TR, mean duration 11 sec).
Design specifications	Each participant received 5 experimental task runs (each 7 min 40 sec in duration) of 40 trials each (20 OLD and 20 NEW trials) for a total of 200 trials (100 OLD and 100 NEW). Trials ranged in 8-14 seconds in duration in multiples of the TR with a uniform distribution (8, 10, 12, 14 seconds), mean trial duration = 11 seconds.
Behavioral performance measures	Each picture naming trial was scored for correctness by the experimenter, with response times and MRI data included in analyses for correct trials only. Responses longer than 2 seconds were also excluded (mean naming time is typically 700-900 msec). Participants performed between 85-90% correct on the average.

Acquisition

Imaging type(s)	functional with a structural included for common volume-based registration to Talairach & Tournoux atlas.
Field strength	3T
Sequence & imaging parameters	Images were acquired with a General Electric Signa HDxt 3.0T scanner (GE Healthcare) using an 8-channel receive-only head coil. A high-resolution T1-weighted anatomical image (MPRAGE, magnetization-prepared rapid gradient-echo) was obtained for each participant (124 axial slices, 1.2 mm slice thickness, field of view = 24 cm, 224 x 224 acquisition matrix). Functional (T2*-weighted) images were acquired using a gradient-echo echo-planar imaging (EPI) sequence [Array Spatial Sensitivity Encoding Technique, ASSET, acceleration factor = 2, TR = 2000 ms, TE = 27 ms, flip angle = 60 deg, 40 sagittal slices (3.5 mm slice thickness), field of view = 216 mm, 72 x 72 acquisition matrix, voxel resolution = 3.5 x 3.0 x 3.0 mm^3].
Area of acquisition	Whole-brain

Diffusion MRI ☐ Used ☒ Not used

Preprocessing

Preprocessing software	Preprocessing is provided in detail in the methods. It is copied here for convenience: Preprocessing utilized the AFNI software package60, applying steps in the following order: 1) removal of the first 3 TRs to allow for T1 equilibration; 2) 3dDespike to bound outlying time points per voxel within 4 standard deviations of the time

series mean; 3) 3dTshift to adjust for slice acquisition time within each volume (to t=0); 4) 3dvolreg to align each volume of a run's scan series to the first retained volume of the first run; 5) each scan was then spatially blurred by a 6-mm Gaussian kernel (full width at half maximum) and divided by the voxelwise time series mean to yield units of percentage signal change. De-noising of each scan then utilized the ANATICOR nuisance regression approach34,61. White matter and large ventricle masks were created from the aligned MPRAGE scan using Freesurfer62, and a large draining vein mask was created from a standard deviation map of the volume-registered EPI data (from step 4 above). All masks were resampled to EPI resolution and eroded by 1 voxel to prevent partial volume effects with gray matter voxels, and the related nuisance time series were calculated on the volume-registered data just prior to spatial blurring (after step 4 and prior to step 5 above). Nuisance regression for each voxel was performed on the spatially blurred volume-registered data (after step 5 above), and the regressors consisted of: 6 head-position parameter time series (3 translation, 3 rotation), 1 average eroded ventricle time series, 1 "localized" eroded white matter time series (averaging the time series of all white matter voxels within a 20-mm radius sphere), 1 eroded draining vein time series, 8 Retroicor time series (4 cardiac, 4 respiration) calculated from the cardiac and respiratory measures taken during the scan63, 5 Respiration Volume per Time (RVT) time series to minimize end-tidal CO2 effects following deep breaths64, and the first 3 principal component time series calculated on a union mask of the nuisance tissues (white matter, ventricles, draining veins) (aCompCor regressors65,66). Prior to regression, all nuisance time series were detrended by a 4th-order polynomial function to remove slower scanner drift and drift in head position, with the de-noised residuals detrended in the same manner during regression. After regression, de-noised residual time series were transformed to standardized anatomical space (Talairach-Tournoux) for task analyses at a resolution of 3 mm3 isotropic.

Normalization

Voxel time series were normalized by voxelwise mean to units of percentage signal change (which adjusts for differences in shim and field strength per participant).

Normalization template

Volume-based analyses were conducted in Talairach-Tournoux atlas space.

Noise and artifact removal

This is described in detail above in the section on preprocessing for level-I artifact removal (using a mix of ANATICOR and aCompCor nuisance regressors). This involved regression of the head motion parameters, as well as regressors derived from the cardiac and respiratory cycles, which were recorded simultaneously for all participants. Additionally, participant-level covariates for each condition were included during group-level analyses using linear mixed effects modeling (3dLME): the grand-average correlation over all voxel time series (GCOR, Saad et al., 2013; Gotts et al., 2013). This covariate will contain any residual global artifacts such as head motion and respiration that persisted beyond the level-I denoising.

Volume censoring

Rather than censoring high-motion timepoints, we confirmed that each run had relatively low transient motion on the average (< 0.3 mm/TR, in terms of Framewise Displacement). This assures that the residual head-motion will stay in the relatively linear range of motion to functional connectivity, allowing the participant-level covariates in the group-level analyses to function appropriately (see Gotts et al., 2020, Neuroimage, supplementary materials for discussion). This criterion led to the exclusion of 1 participant in the Overt Naming task (2/5 runs with > 0.3 mm/TR motion; mean motion over all runs > 0.3 mm/TR).

Statistical modeling & inference

Model type and settings

Most analyses (voxelwise and region-of-interest-wise) utilized linear mixed effects models. Linear mixed effects (LME) models (using AFNI's 3dLME) were constructed whose dependent variables were the functional/effective connectivity measures (or peak times for Facilitation model tests, spatial correlation measures for Sharpening model tests) in each experimental condition. Task, Repetition, Primeability, and their interactions were included as fixed effects. The global level of correlation among all brain voxels, GCOR, was included as a nuisance covariate in order to model any residual motion and/or breathing artifacts present after the nuisance regression. Participant was treated as a random intercept.

Effect(s) tested

This is provided in detail in the methods and results sections for each test. In general, we tested for main effects of Repetition and interactions between Repetition and Primeability (all F-tests in LME models). Higher-level interactions of these effects with Task (Overt versus Covert Naming) were also evaluated to confirm that there were no significant interactions that would undermine generalizability across tasks (also F-tests). Contrasts of OLD/NEW for effects with significant F-tests were performed as Z-tests (from 3dLME in AFNI).

Specify type of analysis: ☐ Whole brain ☐ ROI-based ☒ Both

Anatomical location(s)

GLM analyses for each task separately (Overt Naming and Covert Naming) identified regions showing repetition suppression at stringent levels of FDR correction (e.g. q<.00016 in task responses; q<.00006 in RS/RE tests). A conjunction of voxels across task corresponded to voxels for which an effect replicated across tasks. The maximum statistic of the combined data (Overt+Covert) then identified the peak coordinate of RS in the GLM-based ROIs. For ROIs showing Repetition X Primeability interactions in functional connectivity, the coordinates corresponded to the location of the maximum F-statistic for that interaction (in the connectedness test for the right TP ROI, in the seed-based tests for the ACC, right fusiform, right putamen and right STG ROIs). All ROIs were then sampled with 6-mm radius spheres centered at the peak coordinates.

Statistic type for inference
(See Eklund et al. 2016)

GLM analyses were voxelwise, and all initial functional connectivity analyses were voxelwise using whole-brain connectedness, with follow-up voxelwise seed-based tests. ROI-based analyses were subsequent to these.

Correction

GLM analyses were all corrected by FDR (although all were also corrected by cluster-size). Functional connectivity analyses were corrected by cluster size. Gaussianity was not assumed of the spatial distribution of effects (following Eklund et al., 2016), and used monte carlo simulations based on the empirically measured spatial autocorrelation function in AFNI (the acf option in AFNI's 3dFWHMx and 3dClustSim functions). All voxelwise statistical thresholds were set at P<.001, for which the acf methodology has been shown to control for the false positive rate at P<.05 or less (Cox et al., 2017, in Brain Connectivity). Since two effects were searched for whole-brain (Repetition and Repetition X Primeability), the corrected alpha was set at P<.025 (.05/2) so that the full familywise alpha would be P<.05. This corresponded to a cluster-size theshold in the group-

analysis mask of k=25. The estimated corrected P-value of the TP seed was P<.02, and all clusters from the seed test had corrected P-values of P=.01 or less.

All matrix tests, correlations between brain measures and priming, as well as multiple comparisons in the tests of the Facilitation and Sharpening models, were corrected by FDR.

Models & analysis

n/a	Involved in the study
☐	☒ Functional and/or effective connectivity
☒	☐ Graph analysis
☒	☐ Multivariate modeling or predictive analysis

Functional and/or effective connectivity

Fisher-z transformed Pearson correlations were used in functional connectivity analyses. These served as the inputs to the structural equation modeling (SEM) analyses used in effective connectivity (implemented in 1dSEM and 1dSEMr in AFNI).
